# Landscape dynamics revealed by luminescence signals of feldspars from fluvial terraces

**DOI:** 10.1038/s41598-019-44533-4

**Published:** 2019-06-12

**Authors:** Stéphane Bonnet, Tony Reimann, Jakob Wallinga, Dimitri Lague, Philippe Davy, Aurélien Lacoste

**Affiliations:** 1 Géosciences Environnement Toulouse (GET), Université de Toulouse, CNRS, IRD, UPS Toulouse, France; 2 Soil Geography and Landscape group & Netherlands Centre for Luminescence Dating, Wageningen University, Wageningen, The Netherlands; 3 Univ Rennes, CNRS, Géosciences Rennes, UMR 6118, Rennes, France; 4 E.A. 6293 GéoHydrosystèmes Continentaux, Université de Tours, Tours, France

**Keywords:** Geodynamics, Geology, Geomorphology, Sedimentology, Tectonics

## Abstract

Luminescence signals of quartz and feldspar minerals are widely used to determine the burial age of Quaternary sediments. Although luminescence signals bleach rapidly with sunlight exposure, incomplete bleaching may affect luminescence ages, in particular in fluvial settings where an unbleached remnant signal is commonly encountered in modern alluvium. Here, we use feldspar single-grain post-infrared IR stimulation (pIRIR) dating to show that recent (<11 ka) fluvial terraces of the Rangitikei River (New Zealand) were formed in a context of non-linear incision rate. We relate this pattern to the rapid reinstatement of steady-state incision following the formation of a major, climate-driven, aggradation terrace, causing a phase of accelerated incision. In addition, we show systematic variations in the proportion of unbleached grains in the fluvial sediments over time, mirroring incision rate at the time of deposition. Deposits formed during rapid incision contain fewer bleached grains, which we attribute to large input of unbleached material and limited bleaching opportunities during fluvial transport. This finding demonstrates that the luminescence signals recorded in fluvial terraces not only yield age information, but also inform us on past fluvial transport and ultimately, landscape dynamics.

## Introduction

In the past decades the development of geochronological methods for dating Earth’s surface features^1,2^ has been crucial for dating fluvial terraces, quantifying incision and erosion rates^3–5^ and improving our understanding of the interplay between tectonics and climate in shaping the Earth surface (e.g. ref.^5^). Those based on Optically Stimulated Luminescence (OSL) on quartz or on Infrared Stimulated Luminescence (IRSL) on feldspars allow estimation of the time of deposition and burial of sediments^1,6^, but sometimes give overestimated ages when light-exposure has not been sufficient to reset the luminescence signals prior to burial^7^, a problem that can be significant in fluvial settings. Actually, although luminescence signals bleach rapidly with sunlight exposure^8^, an unbleached remnant signal is commonly encountered in modern alluvium^9–15^. The reduction of the light intensity and spectrum in the water column due to high sediment concentration and turbidity^16–18^ can limit bleaching during fluvial transport, leading for instance to age reversal in stratigraphic sequences (e.g. ref.^19^). Accordingly, poor bleaching evidence in stratigraphic sequences has been used for example to infer hyperconcentrated flows^20^. Moreover, a better bleaching of modern fluvial sediments in the downstream direction is reported^11–15^, indicating that the transport distance is another important factor for bleaching the luminescence signal. Recently, the idea emerged that one could take advantage of this dating limitation by using along-stream bleaching characteristics to quantify suspended sediment transport^14,15,21,22^. Feldspars constitute preferential targets for this purpose because the feldspar IRSL signals bleach at significantly lower rates than the quartz OSL^8,23–25^.

**Figure 1:**
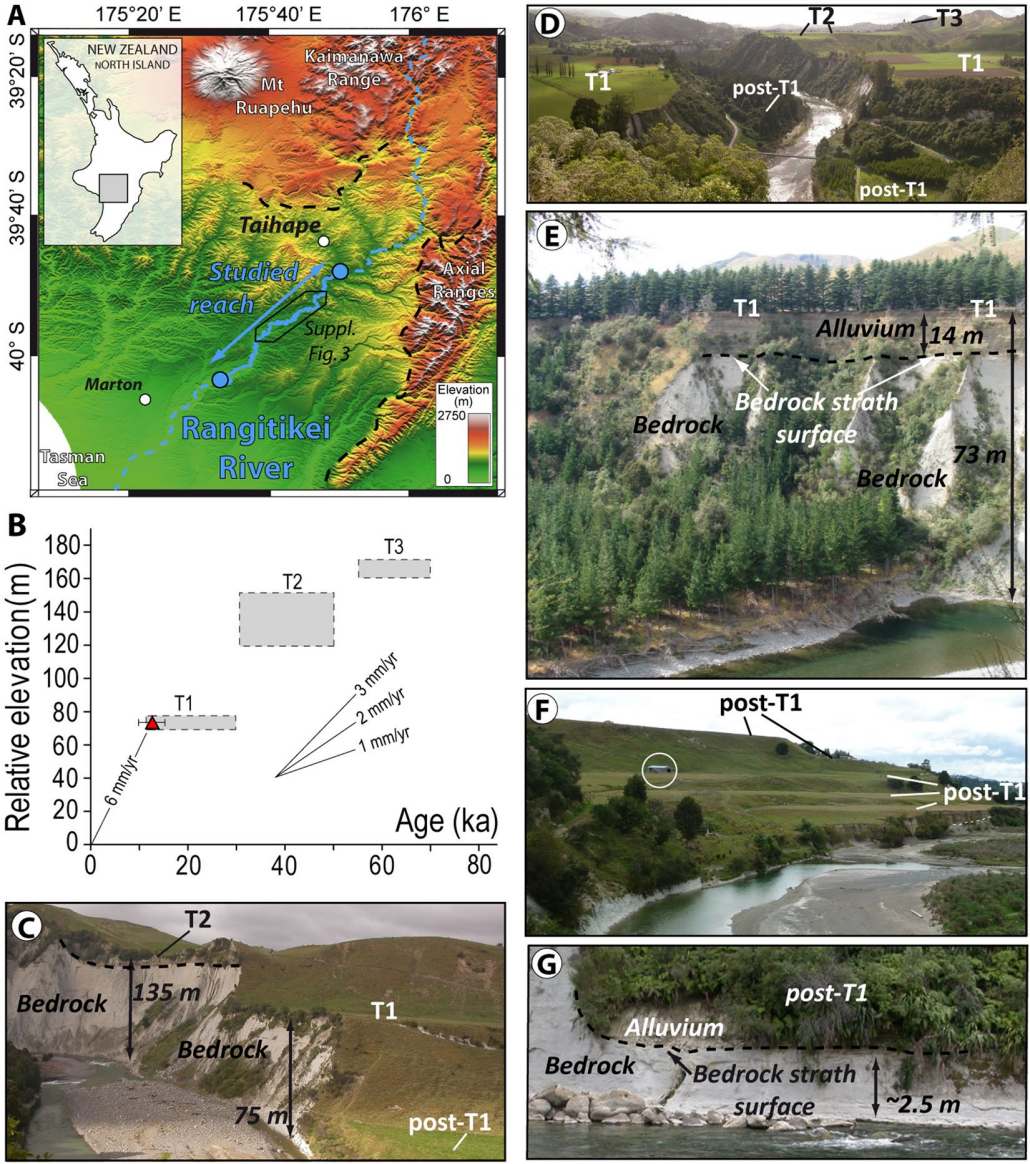
(**A**) Map of the south of North Island of New Zealand showing location of the Rangitikei River (RR; blue line; DEM generated using topographic data from Land Information New Zealand (https://www.linz.govt.nz/; based on LINZ’s data which are licensed by Land Information New Zealand (LINZ) for re-use under the Creative Commons Attribution 4.0 International licence) and ArcGIS 10.4 software (www.ArcGIS.com)). Blue solid circles indicate the upstream and downstream ends of the studied reach where the terraces have been surveyed. Samples for luminescence analysis come from the area demarcated by a thin black line (see Supplementary Fig. 3). Black dotted line shows the approximate boundary of the Plio-Pleistocene marine deposits of the Wanganui Basin, which constitute the bedrock eroded by the RR in our study area. (**B**) Graph of relative elevation of terraces T1, T2 and T3 above the RR *vs*. terrace ages (ages from ref.^29^). Red triangle shows our single-grain BS MAM dating of T1. (**C**) Photograph showing T2 and T1 treads and post-T1 incision of the RR into the bedrock (Plio-Pleistocene shallow marine deposits, in light grey colors). (**D**) Panoramic view of the RR showing the wide area of T1 and less-preserved remnants of T2 and T3. (**E**) Photograph showing thickness of alluvial deposits overlying T1 strath surface. (**F**) Photograph showing post-T1 terraces. Open circle shows a barn for scale. (**G**) Photograph showing alluvial deposits overlying a post-T1 strath surface.

The applicability of feldspar luminescence dating has strongly increased in recent years following development of methods to circumvent anomalous fading^26^ and development of single-grain techniques to investigate the degree of bleaching of a sample and to extract the population of well bleached grains from a heterogeneously bleached sample^27^. We applied here the single-grain post-infrared IR stimulation (pIRIR) IRSL procedure^28^ (see Methods) on feldspars from fluvial terraces to constrain the pattern of fluvial incision and evaluate the concomitant bleaching efficiency of the IRSL signal (Fig. 1). We complement this dataset by acquiring a second dataset of more conventional multigrain IRSL ages on the silt fraction (see Methods) which, in the absence of measurements for individual grains, only permitted determination of mean apparent ages, i.e. ages that overestimate true burial age when the luminescence signal is poorly bleached.

## Landscape and Fluvial Terraces of the Rangitikei River (New Zealand)

In the North Island of New Zealand (NINZ; Fig. 1A), the Rangitikei River (RR) is downcutting into bedrock in response to a rock uplift of ~1.5 to 2.0 mm/y^29^. In the study area, this uplift drives exposure and erosion of a ~750 m-thick sequence of Plio-Pleistocene marine deposits^30^, which constitute the bedrock of the RR in the study area. The incision of the RR has been interrupted by periods of climatically-controlled aggradation as witnessed by strath surfaces covered by fluvial deposits forming fluvial terraces, in some cases covered by loess deposits^31–33^. Three major terraces are preserved in the study area (Fig. 1C,D), the most recent and widespread one, T1, being well-preserved at +75 m above the actual river bed. Its strath surface is covered by up to 15 meters of pebbly alluvium (Fig. 1E) deposited in a braiding setting^31,32^ (Supplementary Fig. 1). Existing tephrostratigraphy, IRSL and radiocarbon ages on this terrace and similar terrace on NINZ, show that T1 developed from 30 to 11 ka^29,33^. Above T1, we observed some remnants of older terraces (T2 and T3: ref.^29^) along our studied reach (Fig. 1C,D). Terraces T1–T3 define a trend in an age-elevation plot (Fig. 1B) that is consistent with a long-term incision rate of 1.5 to 2.0 mm/yr, indicating that incision balances uplift over the long-term (~100 ka).

**Figure 2:**
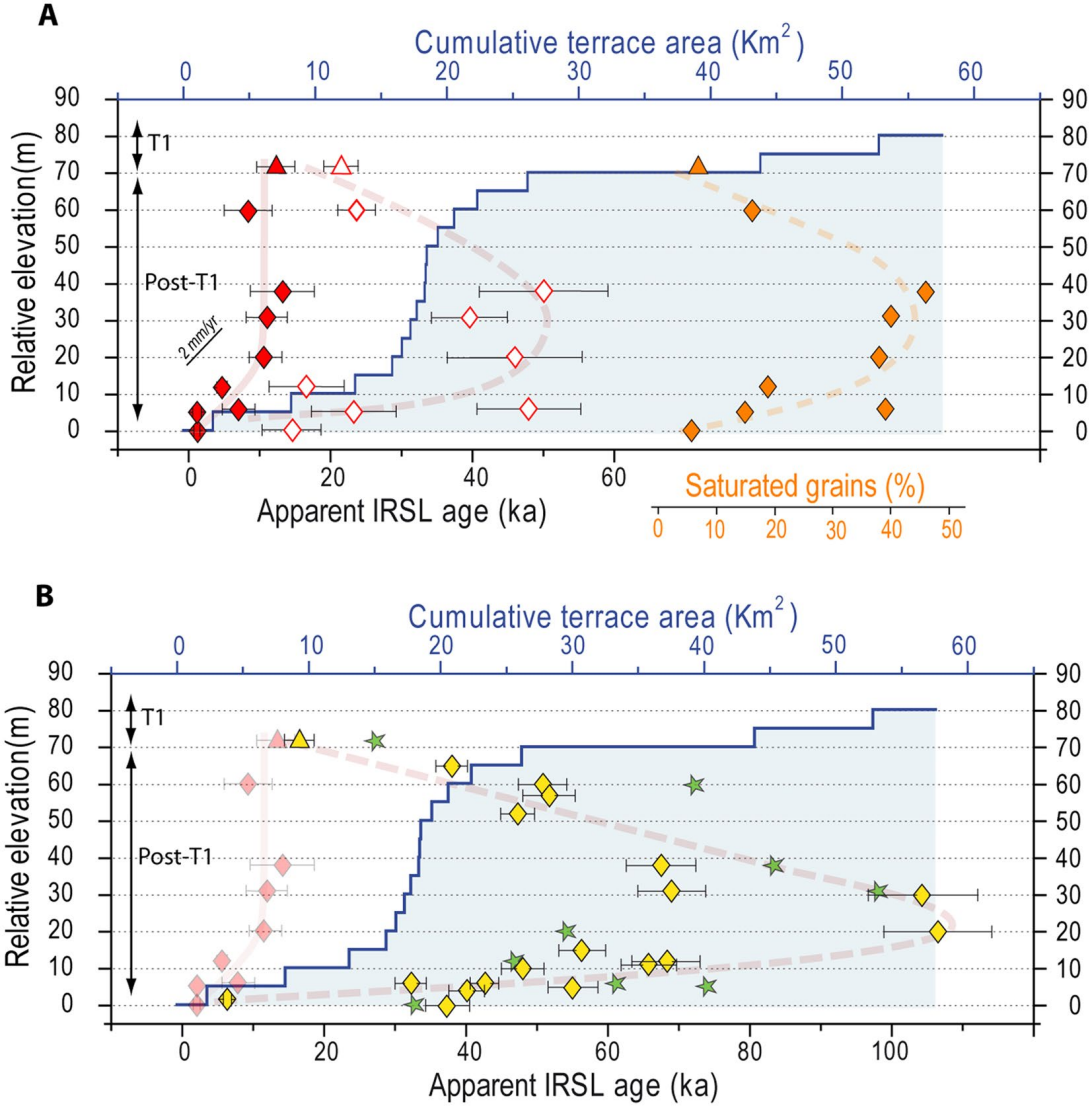
Geometry of T1 and post-T1 terraces and apparent luminescence ages. Blue line and shading show the cumulative area of post-T1 and T1 terrace remnant treads for relative elevation increments of 5 m. Terraces below 70 m are post-T1. Symbols indicate dating results for T1 (triangles) and post-T1 (diamonds). (**A**) pIRIR single-grain dating results. Red symbols are BS MAM (solid) and CAM (open) apparent ages. Orange symbols show the percentage of saturated grains in the samples (see Methods). (**B**) Multi-grain MAAD apparent ages (yellow). Single-grain BS MAM ages (red solid symbols) are also shown for comparison. Green stars indicate age of synthetic multigrain samples (see text).

Along the investigated reach, the RR has incised 75 meters-deep gorges into T1 deposits and the underlying bedrock (Fig. 1C,D). This downcutting has left a flight of narrow and minor unpaired cut terraces (Fig. 1F) that consist of a thin (<3 m) alluvial mantle resting on a strath surface (Fig. 1G). These terraces, henceforth referred to as post-T1^29^ are difficult to correlate along the length of the river and are not homogeneously distributed between T1 and the modern river bed (Fig. 2; see also Supplementary Fig. 2). On a dataset of 299 post-T1 terrace remnants mapped over a ~50 km-long reach of the RR where T1 is at a constant elevation of ~75 m above the valley floor (see Methods), the majority (198) of post-T1 are observed between relative elevations of 0 and +20 m, over a cumulative area of ~15 km^2^, whereas fewer (87) post-T1 are observed at greater height, between +20 to +60 m, representing less than 5 km^2^. Near +75 m, 47 remnants of T1 are observed. Although small in number, their cumulated area (31 km^2^) is larger than that of all 299 post-T1 terrace remnants combined (26 km^2^) (Fig. 2A). As distinctly observable in the field (Fig. 1C,F), these data reveal a lack of terrace remnants from the post-T1 incision phase especially in the elevation range of +20 to +60 m, both in terms of number and area. In contrast, many small terraces are present between +20 m and the current valley bottom. This specific distribution of post-T1 terrace remnants could reflect a difference of preservation (older post-T1 terraces being less preserved) or could alternatively be primary, resulting from a change in the ratio of vertical-to-lateral erosion during post-T1 incision^34,35^. Indeed, the formation of a terrace requires valley widening by lateral erosion, a process that can be inhibited by fast vertical incision^34,35^. To verify this later assumption we constrain the post-T1 incision pattern from single grain pIRIR ages of post-T1 terraces.

## Rate and Pattern of Fluvial Incision

**Figure 3:**
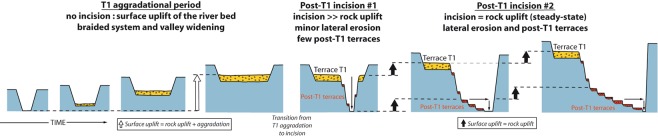
Conceptual model (not to scale) illustrating how the uplift of a river bed during a climatically-induced period of fluvial aggradation impacts its subsequent incision. Strong disequilibrium at the transition from aggradation to incision drives a first period of ultra-fast incision into bedrock with minor lateral erosion whereupon river recovers its steady incision with regards to uplift (see main text). Then, the concomitant increase in lateral erosion promotes river migration and the formation of terraces.

Luminescence is used to date the time of burial of deposits by estimating two quantities. Firstly, the estimate of radiation dose received by the mineral grains since the last deposition and burial event (Palaeodose). It is obtained from the equivalent dose (D_e_) distribution, which in turn is obtained from a comparison of natural luminescence signals to those induced by laboratory radiation exposure. Secondly, the yearly radiation dose during burial [dose rate] is determined from activity concentrations of natural radionuclides in the sample. By dividing the palaeodose by the dose rate, the time elapsed since deposition and burial of the mineral grains is obtained. Here, we sampled sandy lenses within alluvium overlying T1 and post-T1 straths and we applied the recently developed post-infrared IR stimulation (pIRIR) procedure to these samples (Methods), which allows minimizing problems related to feldspar specific athermal signal instabilities^26^. We used a single-grain pIRIR procedure similar to that of ref.^28^; the relatively low preheat and readout temperatures mitigates age overestimations due to insufficient resetting of luminescence signals and it was previously successfully applied for dating Holocene alluvial deposits^36^. For nine samples (1 T1 and 8 post-T1; Supplementary Table 1; Figs 3 and 4), 300 feldspar grains (125–200 or 180–250 μm) were analyzed. We made use of the 2*D_0_ criterion^37^ to distinguish between grains below and above luminescence signal saturation. Ages obtained with Central Age Model^38^ (CAM, providing a weighted mean of single-grain D_e_ estimates) were highly inconsistent with stratigraphic order, with oldest ages obtained for the middle strath terraces. Moreover, the single-grain D_e_ distributions were scattered and right-skewed for all samples (Supplementary Fig. 5), with largest scatter observed for the samples in the middle of the sequence, which also yielded the highest CAM ages. This combined evidence indicates that light exposure was too limited to completely reset pIRIR signals in all grains prior to deposition. To obtain burial age estimates, the bootstrapped Minimum Age Model^38,39^ (BS MAM) was therefore applied, which selects the lower part of D_e_ distribution, assuming that part of the grains is well-bleached at the time of deposition. The BS MAM has been shown to provide accurate estimates of burial dose for both well-bleached and heterogeneously bleached samples, provided that the expected over-dispersion for well-bleached samples (sigma b) is estimated correctly^40^. We complement this dataset by analyzing the luminescence of large, multi-grain, aliquots from the fine-grain fraction (4–11 µm) of the samples (Methods). Due to intra-aliquot signal averaging, this second dataset only permitted determination of mean apparent ages from the bulk signal of well-bleached and partially-bleached grains, which have been calculated using the multiple aliquot additive-dose (MAAD) protocol (e.g. ref.^41^). This approach was applied to the 9 single-grain samples plus 10 additional post-T1 samples (Supplementary Table 2 and Fig. 3).

The single-grain D_e_ distribution of T1 and post-T1 terraces provide evidence for heterogeneous bleaching (Supplementary Fig. 5) and ages based on CAM strongly disagree with geomorphic evidence of stratigraphic order. This combined evidence suggests that the BS MAM provides the best estimate of the depositional ages. The BS MAM approach for single-grains of feldspar yields a T1 age of 12.4 ± 2.7 ka (Supplementary Table 1), slightly younger than previous age inferences for the RR T1 (~18 ka^29,32^) but in agreement with the minimum ages for the correlative terrace in neighboring catchments^33^ (11.3 ± 0.8 and 13.2 ± 0.9 ka). The age and elevation of T1 indicate a mean post-T1 incision rate of about 6 mm/yr, significantly higher than the long-term average incision rate, as observed in the case of most NINZ rivers^29^. We consider BS MAM ages of post-T1 terraces to detail the actual pattern of post-T1 incision (Fig. 2A), although this analysis is complicated for post-T1 terraces between +60 and +20 m (Supplementary Table 1) due to uncertainties in age estimates resulting from highly-scattered D_e_ distributions lacking a clear leading edge **(**Supplementary Fig. 5). However, best-estimate depositional ages (BS MAM) for these terraces are very similar, all around 11 ka. In contrast, BS MAM ages of post-T1 located between +20 m and the present river bed are significantly younger (Fig. 2A). Thus, these ages do not support a constant incision rate into the bedrock since T1 abandonment (Fig. 1B), but rather a non-steady pattern characterized by an initial fast incision phase followed by a much slower incision period (Fig. 2A). This pattern is consistent with previous inferences made for a tributary of the Waipaoa River (NINZ)^42^. The range of elevations where dating indicates a slowing down of the incision rate coincides with the occurrence of many post-T1 treads (Fig. 2A) whereas very few exist above, where we document a fast incision. These observations suggest that the heterogeneous distribution of post-T1 reflects the decrease in vertical incision and a concomitant increase in lateral erosion. If this is the case the evolution of the RR likely constitutes a field evidence of the hypothesis that incision rate controls the ratio of vertical to lateral erosion^34^, with high incision rates preventing lateral erosion and the formation of terraces.

For the period where a lessening of the incision rate is observed, age-elevation data are consistent with a mean incision rate of about 2 mm/yr, similar to the long-term incision and uplift rates. This suggests that the RR recovered a steady-state incision with regard to uplift after the initial episode of fast incision. We propose that this non-steady pattern of post-T1 incision is the consequence of a delayed incision with regard to uplift during the formation of T1 (Fig. 3). T1 shows evidence for fluvial aggradation while the RR was braided^31,32^ (see also Supplementary Fig. 1). During this phase, vertical bedrock erosion was inhibited due to the cover effect of the thick layer of alluvium^34,43,44^ but valley widening could occur by lateral erosion^34,45^. In a context of continuous rock uplift, any hiatus of incision would result in surface uplift of the river bed^43^, generating a disequilibrium. Here, considering a rock uplift rate of 1.5–2.0 mm/yr that is not balanced by fluvial incision during the 15 to 20 ka time span of aggradation over T1^29^, and deposition of 15 m-thick alluvium over the T1 strath surface (Fig. 1E), we estimate a streambed surface uplift of 37 to 55 m during the formation of T1, which matches well with the observed depth of fast incision after T1 abandonment. We propose that the non-steady state incision is a response to the riverbed surface uplift and disequilibrium at the end of T1 aggradation period, with an initial period of fast incision to regain equilibrium, followed by a much slower incision corresponding to the steady-state incision rate imposed by uplift (Fig. 3).

## Heterogeneous Bleaching of the Luminescence Signal During Non-Linear Incision of the Rangitikei River

For most samples, there are large discrepancies between BS MAM, CAM, and MAAD apparent ages (Fig. 2). All multi-grain based apparent ages are systematically older than the single-grain BS MAM ones, as would be expected for heterogeneously bleached deposits. Remarkably, the degree and pattern of age overestimation (CAM and MAAD vs. BS MAM) as well as the percentage of saturated, and thus unbleached, grains (Fig. 2A; Methods), all show a clear relationship with sample elevation, both peaking for the 20–60 m elevation interval reflecting the phase of rapid incision. This is particularly clear for the 18 post-T1 MAAD apparent ages that depict a boomerang-shape pattern (Fig. 2B) with older apparent ages, about 10-times the BS MAM ones, for samples coming from terraces at +20/+30 m. A similar pattern is observed for the percentage of saturated grains in the single-grain data (Fig. 2A).

The degree of age overestimation by CAM and MAAD methods is minimal for T1 (Fig. 2). Accordingly, a low percentage of grains with luminescence signals near saturation (‘saturated grains’; 7%; Supplementary Table 1), i.e. of unbleached or infinitively old grains, is observed for this sample. It indicates good bleaching of the vast majority of particles while transported in this braided river system, under aggrading conditions. We relate these good bleaching conditions to frequent exposure of the particles to sunlight during repeated cycles of transport and deposition in the braided system, and relatively long transport distances. In contrast, the large difference between CAM and single grain BS MAM ages of post-T1 terraces (Fig. 2A) is due to a significant proportion of grains that were unbleached or not fully bleached at the time of deposition, and that are mixed with the bleached grains. This finding is supported by the high percentages of saturated grains (~40%) for those samples (Fig. 2A). To further verify this finding for multi-grain MAAD data and to make CAM ages directly comparable to MAAD ones, we generated synthetic multiple-grain ages from our single-grain data, by summing the pIRIR signal of 100 single-grains for each sample (Supplementary Table 3). These synthetic multiple-grain ages (Fig. 2B) confidently reproduce the MAAD ages, which implies that, as for CAM ages, overestimation of MAAD apparent ages results from the presence of unbleached grains at the time of deposition in the samples. Then, the trend depicted by MAAD ages (Fig. 2B) also reflects a mixing curve between unbleached and bleached grains, as for CAM ones, with variable proportions during post-T1 incision. As river terraces in the studied area are not old enough to explain the oldest MAAD apparent ages as well as the omnipresence of grains with saturated signals, we deduce that the main source of unbleached grains is bedrock. Mass wasting processes are currently very active on the sides of the RR canyon^46^ and are thus suitable mechanisms for feeding the river with old unbleached grains, in addition to fluvial bedrock incision itself.

Our data indicate that an increasing fraction of old and/or unbleached grains were progressively transported and incorporated into post-T1 fills during the fast incision period, and that this fraction decreased when the incision rate slowed down. To the best of our knowledge, such a striking dependency between landscape dynamics and the luminescence signals has never been documented before. We discuss two possible mechanisms for explaining these observations. The first is that the light intensity and spectrum in the water column depend on turbidity^16–18^. Hence, it could be expected that variation in the proportion of unbleached grains results directly from variation in sediment concentration in the river driven by incision rate. If true, this finding would offer the unique opportunity to estimate the past turbidity of the river. However, experiments^18^ show that the bleaching of particles is actually very limited during transport in a turbulent flow, even for low sediment concentrations (0.01 g/l). Moreover the idea emerges^21^ that bleaching may take place predominantly during periods of subaerial exposure in channel bars rather than during transport in the flow^47^. This provides support for a second mechanism, where the frequency of subaerial exposure of particles in channel bars prior to deposition and preservation in a fluvial terrace explains the dependency of bleaching on incision rate. Considering an initial stock of unbleached particles, their bleaching due to successive exposures on the floodplain between transportation events, before trapping, will be greatly reduced in a context of fast incision. In that scheme, a low incision rate will conversely enhance the exposure of the particles and ultimately their bleaching. We consequently interpret the increasing trend of poor bleaching and age overestimation as the result of a supply of unbleached grains to the river due to the collapse of valley walls of the RR canyon^46^ driven by the fast incision, coupled to a decreasing efficiency of bleaching because of low recycling probability of the grains in the floodplain before their trapping. As evidenced by the development of post-T1 terraces, the decrease in incision rate coincides with an enhanced lateral erosion and migration of the RR, and then potentially with a widening of its floodplain, which also likely promotes the sub-aerial exposure and bleaching of the particle. Lateral erosion likely also promoted the collapse of valley walls. Thus we propose that decreasing trend of age overestimation results principally from more frequent sub-aerial exposure of the particles in the floodplain due to the lateral divagation of the river and the lessening incision rather than from the decrease in the input of unbleached grains to the river.

Our study demonstrates that the variability of the IRSL signal measured in feldspars from fluvial terraces is closely linked to landscape dynamics. We show that this variability, and the related overestimation of mean luminescence ages, result from a mixing between unbleached and bleached grains with a variable proportion that is tightly linked to fluvial incision rate. We propose that incision rate influences the sunlight exposure and thus magnitude of bleaching of the particles by controlling the frequency of remobilization of the grains during successive cycles of transport and deposition and by controlling the lateral divagation of the river in the floodplain. Beyond dating, this study highlights an emerging capability to use luminescence signals, i.e. intrinsic properties of mineral grains, to extract information on fluvial transport^14,15,21,22^, and ultimately on landscape dynamics.

## Methods

### Geometry of strath-terraces system

We mapped and surveyed the elevation of T1 and post-T1 treads along a ~50 km-long reach of the RR where T1 is at a constant elevation of ~75 m above the valley floor. We identified and mapped 376 T1 and post-T1 terraces from field work and analysis of aerial photographs and of a 25 m square grid DEM. The elevation of terraces was surveyed on the field using DGPS and laser telemeter (214 terraces) or DEMs estimate (162 terraces) when field measurements were not possible (Supplementary Fig. 2). Comparison between DEM-based and field measurements for 214 terraces out of the 376 mapped shows an underestimation of relative elevations from DEM of ~10 m (Supplementary Fig. 2) that was used to correct DEM-based relative terraces elevations.

### Palaeodose determination/single-grain pIRIR

Single-grain pIRIR samples were analyzed at the Netherlands Centre for Luminescence dating at Wageningen University. We applied the post-IR IRSL (pIRIR) measurement procedure of ref.^26^, with parameters for single-grain feldspar pIRIR measurement as detailed in ref.^28^.We used an automated Risø TL/OSL reader (DA 15) fitted with a dual (red and green) laser single‐grain attachment^48^ and ^90^Sr/^90^Y beta source. The grains were loaded into aluminum single‐grain discs with a 10 × 10 grid of 300 μm grain holes. The feldspar grains were each optically stimulated for 1.68 s with a 150 mW 830 nm IR laser. The IRSL at 50 °C was released prior to the pIRIR single‐grain measurement using an array of infrared LEDs (870 ± 40 nm, ~140 mW/cm^2^) for 100 s stimulation. All feldspar (p)IRSL signals were detected through a LOT/Oriel 410/30 interference filter to select the K‐rich feldspar emission around 410 nm^49^.

A pIRIR protocol (Supplementary Table 4) was employed using a relatively low preheat of 200 °C for 60 s and pIRIR stimulation temperature of 175 °C to reduce unwanted effects of thermal transfer in our young samples^50,51^ and to avoid changes in trapping sensitivity that may be introduced by more stringent heating^52,53^. The relatively low measurement temperature also avoids thermal depletion of signals during the measurement. A net signal was calculated from the signal of the first 0.07 s after the IR laser was switched on, minus the time-normalized ‘background’ signal observed in the last 0.48 s (1.20–1.68 s). All dose estimates were derived by projecting the test dose-corrected natural pIRIR signal on the dose response curve obtained through an exponential fit through the test-dose corrected regenerated dose points. Each dose response curve was forced through the origin. For all experiments only single‐grains with a test dose error of less than 20% and a recycling ratio consistent with unity within 2σ were accepted. Furthermore, all grains displaying non‐monotonically growing dose response curves were rejected from further analysis.

To test the performance of the applied single‐grain pIRIR procedure a few hundred K‐rich feldspar grains of sample RO_04 were bleached for 19 hours using a solar simulator (Hönle SOL2). The grains were subsequently loaded into four single‐grain disc (~400 grains). Two single‐grain disc were directly measured and analysed following the approach detailed above to determine the laboratory residual dose after bleaching. The vast majority of grains show dose values around 1 Gy indicating (i) good bleachability of the pIRIR signal and (ii) a low thermal transfer. The average residual dose was calculated to 1.40 ± 0.99 Gy (n = 107). The remaining two single‐grain discs (~200 grains) were given a beta dose of 32.3 Gy prior to the pIRIR single‐grain measurement. The measured dose distribution is symmetric and centered around 32 Gy (Supplementary Fig. 6). The weighted mean of the single‐grain pIRIR distribution was calculated using the Central Age Model^38^ (CAM). The measured CAM dose is 31.9 ± 0.7 Gy (n = 99). The dose recovery ratio (measured/given dose) derived from this distribution is 0.99 ± 0.02. When the residual dose of 1.40 ± 0.99 Gy is subtracted, the measured/given dose recovery ratio is 0.95 ± 0.04. Both results confirm the suitability of the pIRIR measurement protocol for samples from the Rangitikei catchment. Furthermore, the over‐dispersion value of the dose recovery pIRIR single‐grain dose distribution was calculated^38^. An over‐dispersion of ~18% indicates that the spread in the dose recovery distribution cannot be explained by the associated uncertainties on individual dose values based on counting statistics and curve fitting errors calculated using “Analyst 3.24”^54^. This extra spread caused by intrinsic sources is comparable to dose recovery over-dispersion values reported earlier^28,36^ and needs to be considered when interpreting natural dose distributions.

Additionally, we had to account for the expected extra spread in a well‐bleached dose population that remains unexplained after considering intrinsic sources of scatter. This extrinsic over‐dispersion is caused by extrinsic sources of dose scatter other than poor signal bleaching. The most important of these, at least in this type of environment, is micro-dosimetry; differences in beta-dose rate for different grains due to the non-uniform distribution of radionuclide concentrations in the sediment matrix^54^. We estimate this value to be roughly around 20% (e.g. ref.^28^). Adding in quadrature the intrinsic over‐dispersion of 18% from the dose recovery distribution (see above), thus, the overall expected unexplained scatter in a well‐bleached dose population was estimated to be 27%.

The natural single‐grain pIRIR dose distributions of all nine investigated samples show very large over‐dispersion values (68–150%) indicating that the assigned intrinsic and extrinsic uncertainties (27 ± 10%, see above) are too small to explain the spread in the natural distributions. The additional scatter in single-grain palaeodose distributions is expected to reflect incomplete resetting of pIRIR signals prior to deposition and burial in at least part of the grains. Such heterogeneous bleaching is likely for the samples under investigation, given the depositional environment, and the bleaching properties of pIRIR signals (e.g. ref.^24^). This interpretation is further corroborated by the positive skewness of the distributions (Supplementary Fig. 6), a feature often observed for heterogeneously bleached fluvial deposits (e.g. ref.^7^). For such samples, the true burial dose is reflected by the lower part of the single grain D_e_ distribution, and can be calculated using the bootstrapped version of the Minimum Age Model (BS MAM)^39^. To take into account the expected scatter, we used 27 ± 10% as input parameter (sigma_b) for running the model.

We note that the amount of scattering in the pIRIR single‐grain distributions is very prominent especially when compared with more common quartz single‐grain analysis from other fluvial systems. This was previously reported in ref.^50^ for coastal over‐wash sediments as well as in ref.^36^ for Holocene fluvial samples and can be explained by the lower rate of pIRIR signal resetting compared to the quartz OSL and the feldspar IRSL signal^51^.

We have also monitored the percentage of saturated grains as a proxy for the presents of unbleached grains in the fluvial samples. We made use of the 2*D0 criterion^37^ to differentiate between grains below and above signal saturation. Note that the applied test dose (17.3 Gy) was tailored to accurately determine the palaeodose of the well-bleached grains (LGM and younger with doses <50 Gy) and might have be too small to accurately determine the percentage of saturated grains (with D_e_’s ≪ 200 Gy) such the absolute numbers need to be taken with caution. However, we are convinced that the obtained numbers are meaningful when the samples are compared to each other. The percentage of saturated grains is calculated relative to the total number of luminescent (i.e. light emitting) grains and listed in Supplementary Table 1.

The BS MAM^39^ was applied to the natural single‐grain dose distributions to avoid overestimation of the burial dose by the feldspar grains that were not fully reset at the time of deposition. The final BS MAM palaeodoses (Supplementary Table 1) were derived from the distribution of the 30% intrinsically brightest feldspar grains as only these populations of grains are expected to meet the assumptions with regard to the internal dose rate^28,55^. In order to determine the degree of age overestimation of the single-grain data set we have also derived the weighted mean burial dose by applying the Central Age Model^38^ (CAM) to our pIRIR single-grain dose distribution derived from the 30% intrinsically brightest grains.

It is suggested^50^ that a small additional correction for anomalous fading is required for this kind of pIRIR measurement protocol. Laboratory fading experiments were carried out on two samples (Ro_04 and _18) using multiple grain pIRIR measurements. The average laboratory fading rate of 1.3 ± 0.4 (n = 6) was used for fading correction by applying the fading correction model^56^ to uncorrected pIRIR ages. A similar approach was successfully used previously^28^. The final fading corrected single-grain pIRIR age estimates are listed in Supplementary Table 1.

### Palaeodose determination/Dataset #2 (multi-grain samples)

Multi-grain samples were analyzed at the Luminescence Dating Laboratory, Victoria University of Wellington. The grain size 4–11μm was extracted from the samples in a water-filled (with added dispersing agent to deflocculate clay) measuring cylinder using Stokes’ Law. The samples then are brought into suspension in pure acetone and deposited evenly in a thin layer on 70 aluminum discs (1 cm diameter).

Luminescence measurements were done using a standard Risø TL-DA15 measurement system, equipped with Kopp 5–58 and Schott BG39 optical filters to select the luminescence blue band. Stimulation was done cw at about 30 mW/cm^2^ with infrared diodes at 880Δ80 nm. β-irradiations were done on a Daybreak 801E ^90^Sr,^90^Y β-irradiator, calibrated against SFU, Vancouver, Canada to about 3% accuracy. α-irradiations were done on a ^241^Am irradiator supplied and calibrated by ELSEC, Littlemore, UK.

The paleodoses were estimated by use of the multiple aliquot additive-dose (MAAD) method (e.g. ref.^41^). After an initial test-measurement, 30 aliquots were β-irradiated in six groups up to five times of the dose result taken from the test. Nine aliquots were α-irradiated in three groups up to three times of the dose result taken from the test. These 39 disks were stored in the dark for four weeks to relax the crystal lattice after irradiation.

After storage, these 39 disks and 9 unirradiated disks were preheated for 5 min at 220 °C to remove thermally unstable signal components, and then measured for 100 s each, resulting in 39 shine-down curves. These curves were then normalized for their luminescence response, using 0.1 s short-shine measurements taken before irradiation from all aliquots.

Supplementary Tablesowth curve (β-induced luminescence intensity *vs*. added dose) is then constructed by using the initial 10 seconds of the shine down curves and subtracting the average of the last 20 sec, the so called late light. The shine plateau was checked to be flat after this manipulation. Extrapolation of this growth curve to the dose-axis gives the equivalent dose D_e_, which is used as an estimate of the Paleodose (Supplementary Table 2).

A similar plot for the alpha-irradiated discs allows an estimate of the α-efficiency, the a-value (Luminescence/dose generated by the α-source divided by the luminescence/dose generated by the β-source).

### Dose rate determination

The dry, ground and homogenized sediment samples were encapsulate in airtight perspex containers and stored for at least 4 weeks. This procedure minimizes the loss of the short-lived noble gas ^222^Rn and allows ^226^Ra to reach equilibrium with its daughters ^214^Pb and ^214^Bi.

The samples were counted using high resolution gamma spectrometry with a broad energy Ge detector for a minimum time of 24 h. The spectra were analyzed using GENIE2000 software. The calculations of the external dose rate is based on the activity concentration of the ^40^K nuclide and several nuclides with the Uranium and Thorium decay chain. The contribution of cosmic rays to the external dose rate was calculated according to Prescott and Hutton (1994) assuming instant burial of the samples to present burial depth and taking into account the geographic coordinates and elevation. For the calculation of the internal dose rate for the sand-sized grains a potassium content of 12.5 ± 0.5% and a Rb content of 400 ± 100 ppm was assumed based on refs ^57,58^ respectively. The chemistry of samples and resulting dose rates are listed in Supplementary Tables 1, 2 and 5.

## Supplementary information

Suppl. Figures and Tables
