# Supplementary material for: Landscape dynamics revealed by luminescence signals of feldspars from fluvial terraces
Source: Sci Rep. 2019 Jun 12;9:8569. doi: 10.1038/s41598-019-44533-4 (PMC6561958; doi:10.1038/s41598-019-44533-4)
Supplement: Supplementary file 1 — Suppl. Figures and Tables [file 41598_2019_44533_MOESM1_ESM.pdf]

Supplementary information

Landscape dynamics revealed by luminescence signals of feldspars from fluvial terraces

Stéphane Bonnet, Tony Reimann, Jakob Wallinga, Dimitri Lague, Philippe Davy, and Aurélien Lacoste

Supplementary Table1. Single-grain pIRIR dating results

Sample number	Location		Relative elevation (m)	Palaeodose BS MAM^ (Gy)	Equivalent Dose CAM^ (Gy)	N (grains)*	% of saturated grains □	Dose rate# (Gy/ka)	BS MAM Age (ka) †,§	CAM Age (ka)
	Lat (°S)	Long (°W)								
**RO_04 (T1)**	39.895	175.670	72	32.2 ± 7.4	55.7 ± 5.3	158	7	2.9 ± 0.1	12.4 ± 2.7	21.5 ± 2.4
**RO_18 (post-T1)**	39.843	175.747	60	25.4 ± 10.7	73.9 ± 11.5	93	16	3.5 ± 0.1	8.2 ± 3.6	23.8 ± 2.7
**RO_13 (post-T1)**	39.840	175.768	38	36.7 ± 13.1	137.3 ± 20.4	88	46	3.1 ± 0 .2	13.3 ± 4.5	50.2 ± 9.1
**RO_17 (post-T1)**	39.845	175.754	31	29.8 ± 7.7	107.0 ± 11.9	112	40	3.0 ± 0.2	11.1 ± 2.9	39.7 ± 5.3
**RO_15 (post-T1)**	39.842	175.770	20	30.0 ± 6.9	125.2 ± 22.9	88	38	3.1 ± 0.2	10.9 ± 2.3	46.1 ± 9.5
**RO_10 (post-T1)**	39.908	175.658	12	15.6 ± 2.0	54.0 ± 14.3	67	19	3.6 ± 0.2	4.8 ± 0.7	16.7 ± 5.3
**RO_01 (post-T1)**	39.901	175.671	6	21.2 ± 6.4	139.3 ± 17.8	217	39	3.3 ± 0.2	7.1 ± 2.3	48.1 ± 7.3
**RO_14 (post-T1)**	39.842	175.767	5	3.9 ± 3.0	66.6 ± 16.0	122	15	3.2 ± 0.1	1.3 ± 0.6	23.4 ± 6.0
**RO_20 (post-T1)**	39.809	175.808	0	3.0 ± 1.5	34.5 ± 8.8	143	6	2.6 ± 0.2	1.2 ± 0.5	14.6 ± 4.1

^ Palaeodose is calculated from only the equivalent dose of 30% brightest accepted grains[28,55]

*Number of grains over 300 that gave a measurable luminescence signal. Grain size 125-200 or 212-250 µm

□ % saturated grains relative to the total number of luminescent grains. The $2D_0$ criterion of ref.[37] was used to decide upon saturation

†Dating results from bootstrap Minimum Age Model of ref.[39] (sigma_b 0.27+/-0.10)

§Ages are corrected for fading using the measured average laboratory fading rate of 1.3 ± 0.4 %/Gy

#Conversion factor of ref.[59] was used for dose rate conversion.

Supplementary Table1. Single-grain pIRIR dating results

Supplementary Table2. Multi-grain dating results (MAAD protocol)

Sample number[§]	Location Lat (°S)	Long (°W)	Relative elevation (m)	Equivalent Dose (Gy)	Dose rate[#] (Gy/ka)	Apparent Age (ka)
**RO_04 (T1)**	39.895	175.670	72	49.7 ± 5.6	3.01 ± 0.19	16.5 ± 2.1
RO_03 (post-T1)	39.897	175.671	65	118.0 ± 2.4	3.10 ± 0.17	38.0 ± 2.2
**RO_18 (post-T1)**	39.843	175.747	60	189.1 ± 10.0	3.72 ± 0.15	50,8 ± 3.4
RO_05 (post-T1)	39.898	175.671	57	143.4 ± 5.0	2.78 ± 0.17	51.7 ± 3.7
RO_19 (post-T1)	39.911	175.649	52	200.0 ± 5.8	4.23 ± 0.18	47.3 ± 2.4
**RO_13 (post-T1)**	39.840	175.768	38	223.8 ± 10.6	3.32 ± 0 .18	67.5 ± 4.9
**RO_17* (post-T1)**	39.845	175.754	31	214.6 ± 9.6	3.11 ± 0.17	69.0 ± 4.8
RO_12 (post-T1)	39.908	175.654	30	282.8 ± 8.3	2.71 ± 0.18	104.4 ± 7.7
**RO_15 (post-T1)**	39.842	175.770	20	345.0 ± 13.3	3.24 ± 0.19	106.5 ± 7.6
RO_09 (post-T1)	39.873	175.697	15	193.4 ± 9.4	3.43 ± 0.11	56.4 ± 3.3
**RO_10 (post-T1)**	39.908	175.658	12	272.9 ± 5.0	4.00 ± 0.27	68.2 ± 4.8
RO_11 (post-T1)	39.908	175.658	11	229.4 ± 6.5	3.49 ± 0.18	65.8 ± 3.9
RO_06 (post-T1)	39.875	175.693	10	166.6 ± 7.2	3.47 ± 0.15	48.0 ± 3.0
**RO_01 (post-T1)**	39.901	175.671	6	111.6 ± 2.3	3.47 ± 0.22	32.2 ± 2.2
RO_02 (post-T1)	39.901	175.671	6	150.7 ± 3.5	3.53 ± 0.14	42.6 ± 2.0
**RO_14 (post-T1)**	39.842	175.767	5	187.5 ± 8.6	3.41 ± 0.15	55.1 ± 3.5
RO_08 (post-T1)	39.875	175.698	4	136.4 ± 4.5	3.40 ± 0.18	40.1 ± 2.5
RO_07 (post-T1)	39.875	175.693	1.5	22.3 ± 1.6	3.50 ± 0.18	6.4 ± 0.6
**RO_20 (post-T1)**	39.809	175.808	0	100.7 ± 0.4	2.69 ± 0.22	38.1 ± 3.1

[§]Bold characters indicate samples belonging to both dataset (see Supplementary Table1)

[*]Palaeodose determined by Single Aliquot Regenerative (SAR) method

[#] Conversion factor of ref.[60] was used for dose rate conversion

Supplementary Table3. Multi-grain synthetic ages

Sample number	Equivalent dose / Synthetic aliquots† (Gy)	Synthetic Age (ka) †
RO_04 (T1)	79.6 ± 21.9	27.3 ± 7.6
RO_18	251.8 ± 9.8	72.3 ± 4.3
RO_13	259.2 ± 9.1	83.5 ± 5.4
RO_17	297.1 ± 21.7	98.2 ± 9.0
RO_15	167.0 ± 71.8	54.2 ± 23.5
RO_10	169.3 ± 13.9	46.8 ± 4.9
RO_01	201.2 ± 3.8	61.2 ± 4.1
RO_14	237.0 ± 38.5	74.0 ± 12.5
RO_20	86.7 ± 32.7	32.8 ± 12.6

§indicating the extra spread in the distribution after considering 18% intrinsic (due to experimental sources of uncertainty) and 20% extrinsic (typically found in well bleached distributions) over-dispersion

† Synthetic multi-grain data built by summing the pIRIR signal of 100 single-grains

Supplementary Table 4. pIRIR feldspar single-grain measurement protocol.

Step	Treatment	Observation
1	Regenerative dose (or natural dose in the 1st circle)	
2	60s preheat to 200°C	
3	100s IR stimulation at 50°C	
4	2s Single-grain IR laser stimulation at 175°C	Ln/Li
5	Laboratory test dose	
6	60s preheat at 200°C	
7	100s IR stimulation at 50°C	
8	2s Single-grain IR laser stimulation at 175°C	Tn/Ti
9	40s IR stimulation at 210°C	
10	Repeat step 1-9 for a range of regenerative doses (incl. zero and repeat dose)	

Supplementary Table 5. Chemistry of samples

Sample number	U (µg/g)	U (µg/g)	U (µg/g)	Th (µg/g)	K (%)	Water content
	from ^{234}Th	from ^{226}Ra, ^{214}Pb, ^{214}Bi	from ^{210}Pb	from ^{208}Tl, ^{212}Pb, ^{228}Ac		(%)
RO_01	3.15±0.31	2.91±0.20	2.40±0.27	10.01±0.14	1.95±0.05	20±10
RO_04	1.87±0.18	2.13±0.12	1.76±0.17	7.75±0.10	1.79±0.04	20±10
RO_05	1.70±0.24	1.89±0.16	1.81±0.22	7.73±0.12	1.64±0.04	19±9
RO_06	2.63±0.27	2.39±0.18	2.14±0.25	8.91±0.13	1.91±0.04	12±6
RO_07	2.31±0.20	2.16±0.13	1.90±0.18	8.09±0.10	2.00±0.04	15±8
RO_08	2.34±0.24	2.31±0.16	2.35±0.22	8.38±0.12	1.97±0.04	15±8
RO_09	2.16±0.20	1.72±0.13	1.76±0.18	7.31±0.10	1.95±0.04	7±4
RO_11	2.75±0.28	2.42±0.18	2.22±0.24	9.23±0.13	1.80±0.04	15±7
RO_12	1.91±0.22	1.95±0.15	2.06±0.20	6.35±0.10	1.67±0.04	22±11
RO_03	2.29±0.16	2.11±0.11	1.86±0.13	8.10±0.09	1.61±0.03	16±8
RO_02	2.33±0.26	1.95±0.17	1.70±0.23	7.77±0.12	1.83±0.04	10±5
RO_10	3.48±0.31	2.90±0.20	2.54±0.26	11.51±0.15	2.04±0.05	20±10
RO_13	2.03±0.18	2.33±0.12	1.92±0.15	7.31±0.09	1.92±0.04	16±8
RO_14	2.43±0.31	2.09±0.17	2.06±0.25	7.25±0.13	1.85±0.04	11±5
RO_15	2.16±0.25	1.93±0.14	2.08±0.20	7.34±0.11	1.81±0.04	15±7
RO_17	2.00±0.29	1.98±0.17	1.88±0.23	7.46±0.12	1.80±0.04	16±8
RO_18	2.57±0.27	2.13±0.15	2.19±0.21	8.86±0.13	2.03±0.05	10±5
RO_19	2.82±0.38	3.17±0.22	2.69±0.29	11.22±0.18	1.82±0.04	12±6
RO_20	1.81±0.27	1.93±0.16	1.81±0.22	6.96±0.12	1.78±0.04	28±14

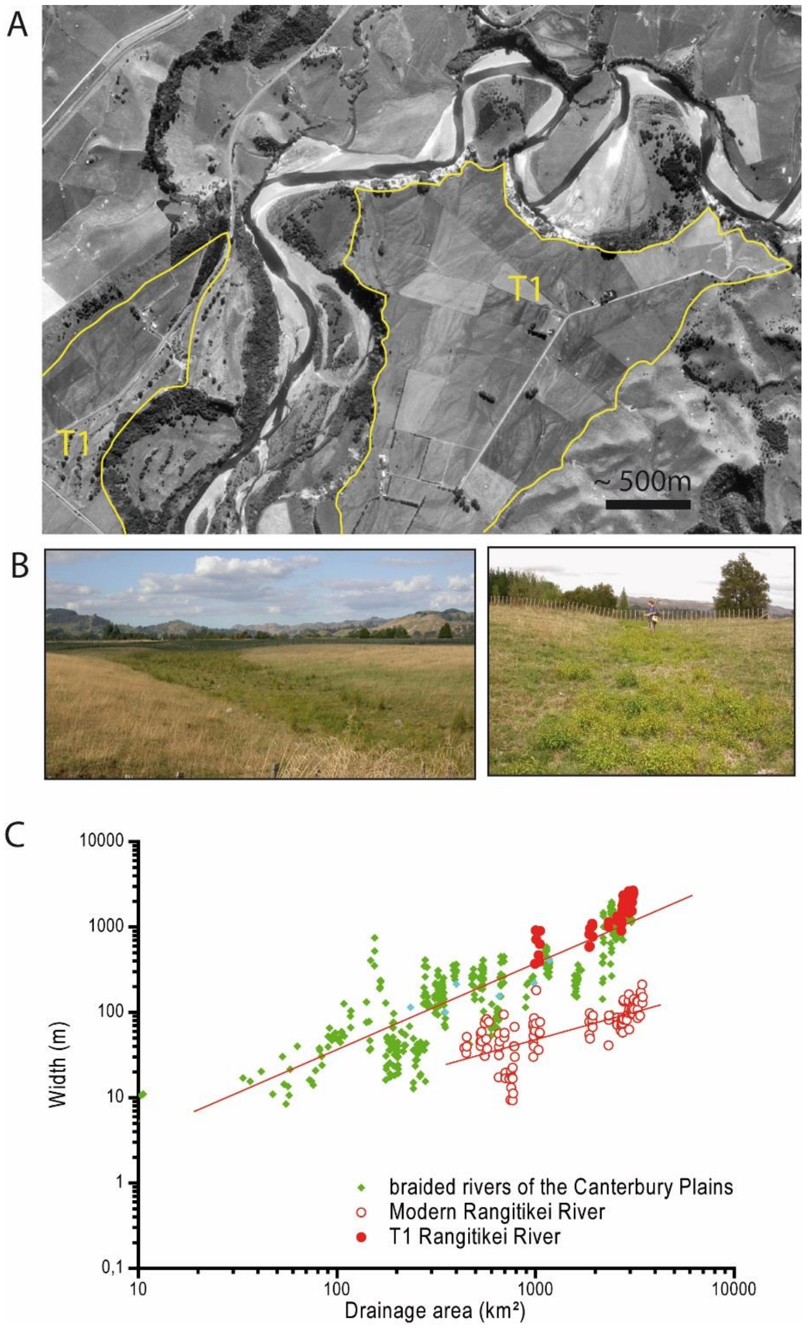

Supplementary Figure 1. Evidence for a braided pattern of the RR during T1 aggradation. A: Example of aerial photograph showing braided paleochannels on T1 tread. Aerial Photograph from Land Information New Zealand (https://www.linz.govt.nz/; based on LINZ's data which are licensed by Land Information New Zealand (LINZ) for re-use under the Creative Commons Attribution 4.0 International licence). Figure generated using ArcGIS 10.4 software (www.ArcGIS.com). B: field view of linear hollows corresponding to braided paleochannels. C: Valley floor width vs drainage area for modern Rangitikei and T1, compared to present-day braided rivers of the Canterbury plain (South Island, New Zealand).

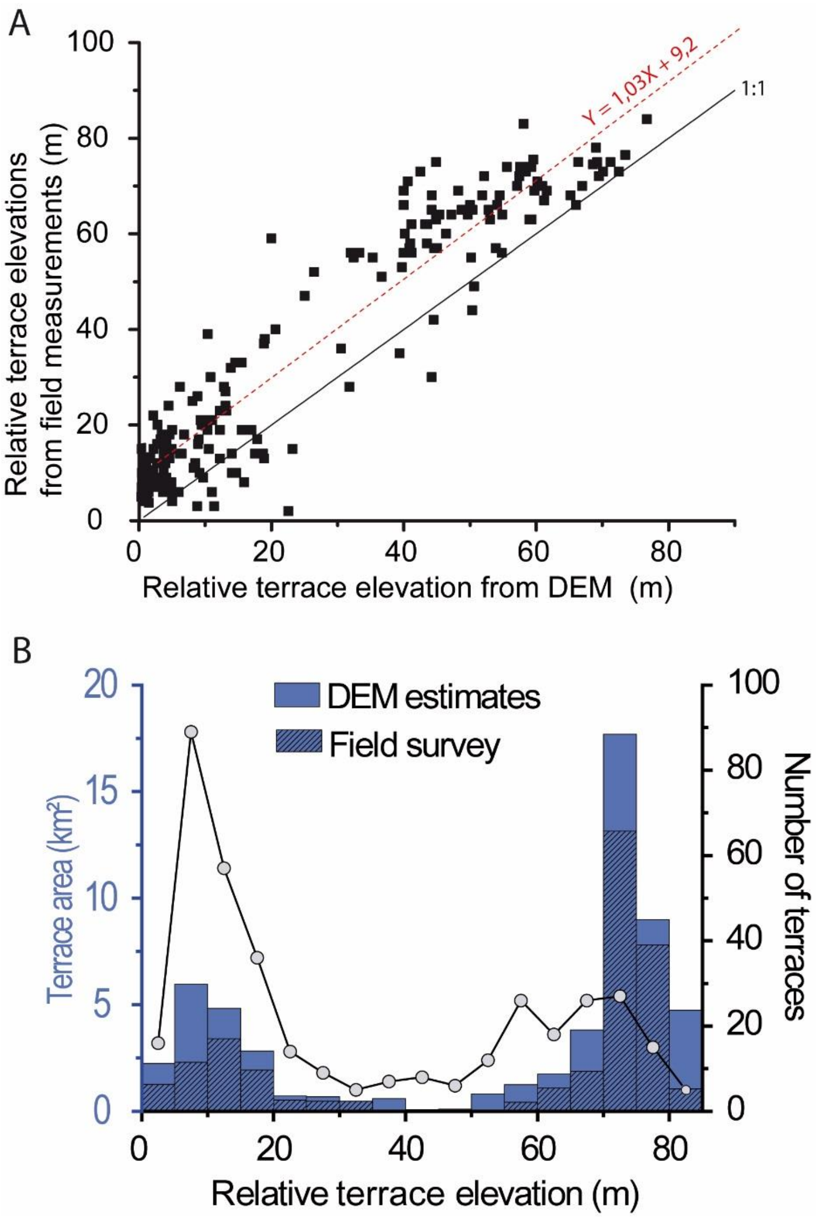

Supplementary Figure 2. Top: relative terrace elevations estimated from DEM vs field measurement. Comparison between DEM-based and field measurements for 214 terraces out of the 376 mapped shows an underestimation of relative elevations from DEM of ~9 m that was used to correct DEM-based relative terraces elevations. The DEM used is a 25 m square grid built from 20 m elevation contours from Land Information New Zealand. Bottom: histogram of terrace area (blue columns) for elevation intervals of 5 m and related number of terraces (grey solid circles).

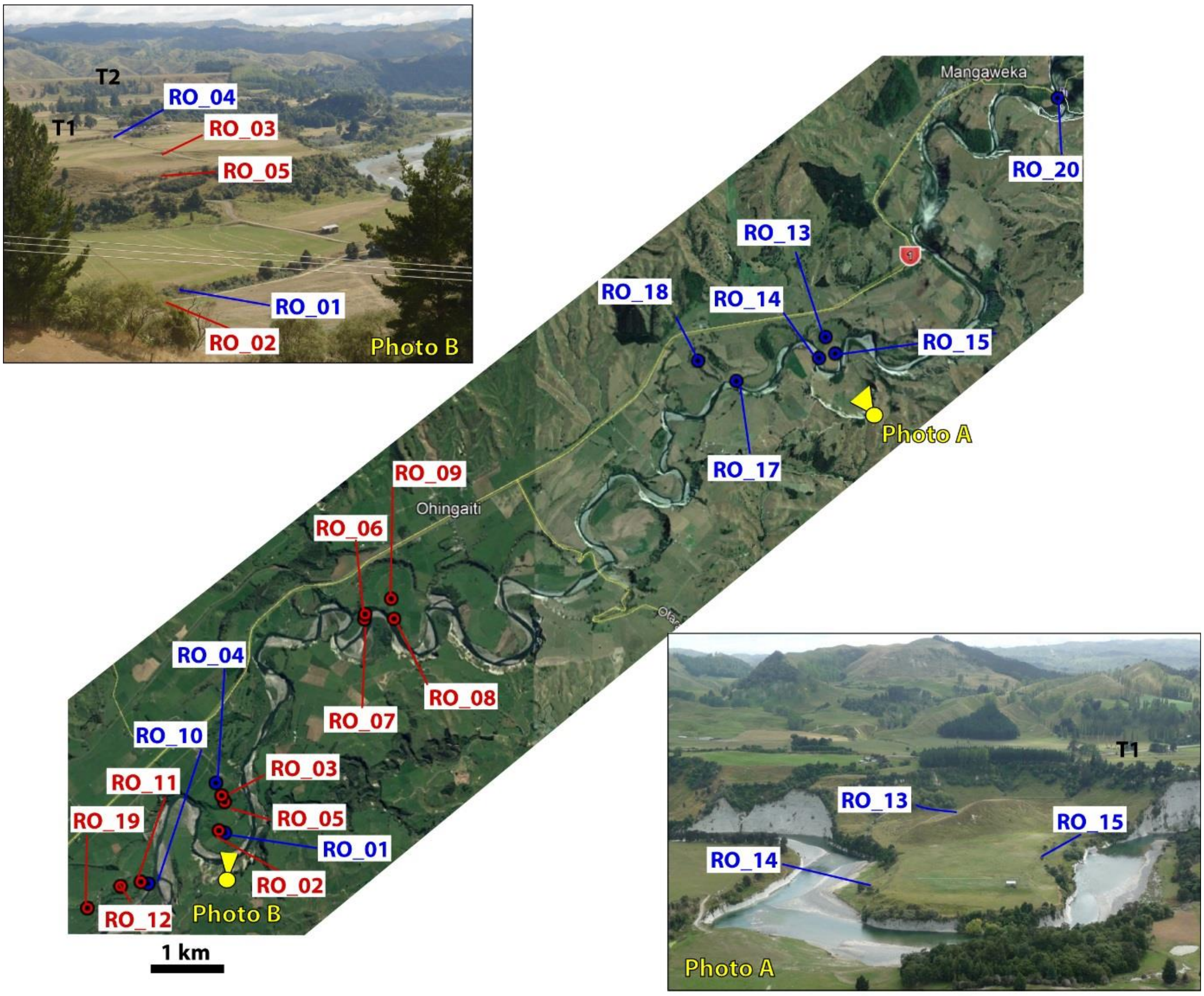

Supplementary Figure 3. Google Earth image (image data: © 2018 DigitalGlobe, image recording 7/11/2018) showing the sampling locations along the studied reach of the Rangitikei River (see location on Fig. 1A; blue: samples with both pIRIR single-grain and MAAD multi-grain data. Red: MAAD multi-grain data only). Field constraints (accessibility, outcrops quality, availability of appropriate materials for sampling, range of relative elevation of terraces) prevent the acquisition of the whole dataset along a single transect in one location and the whole dataset of 19 samples consequently stretches along ~20 km. We endeavored however to reduce the distances between samples in order to minimize some potential diachronicity in post-T1 incision along stream. Photographs A and B (taken by S Bonnet) show landscape and terraces in two sampling places.

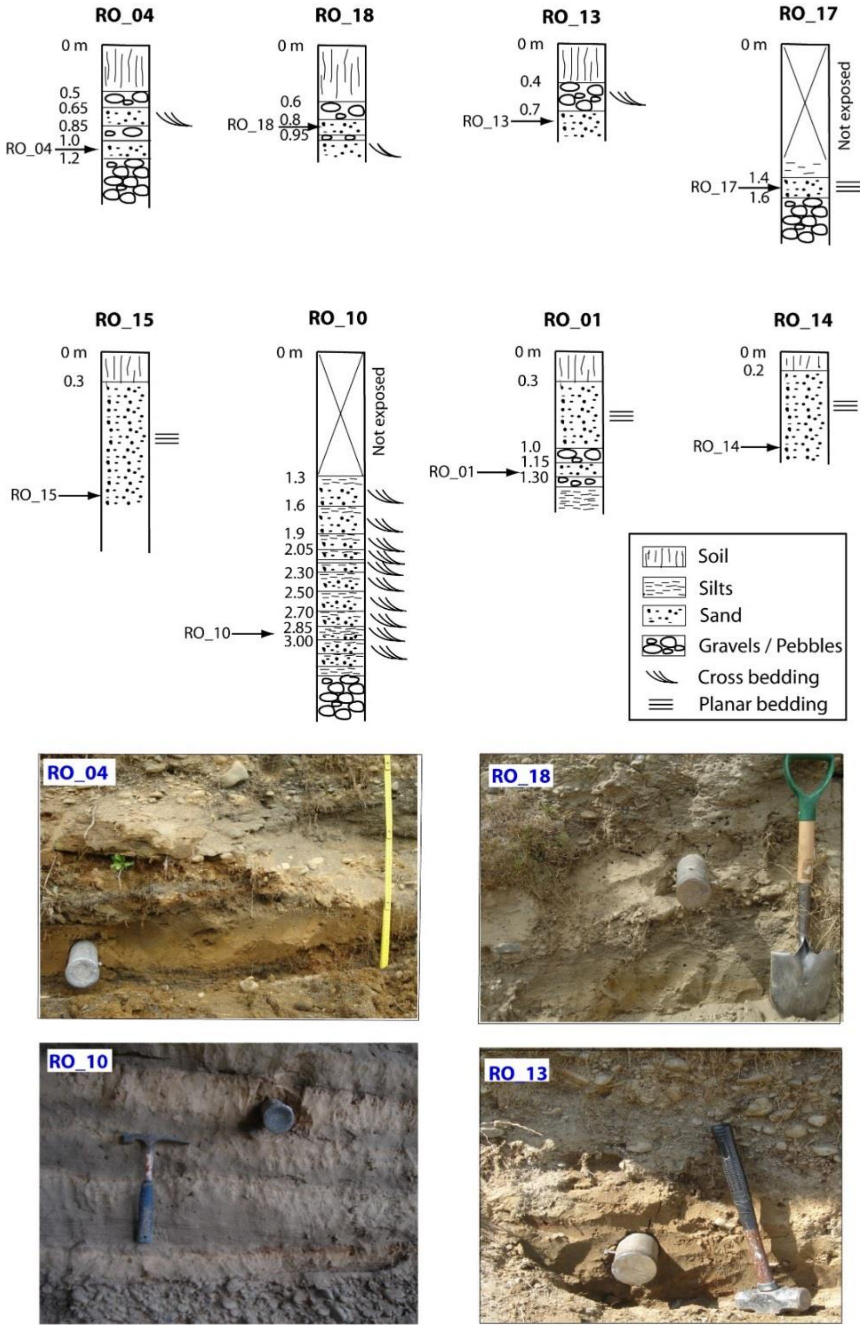

Supplementary Figure 4. Stratigraphic sections for the pIRIR single-grains samples (except for sample RO_20 which corresponds to modern deposits) and photographs of some samples (taken by S. Bonnet). Samples were collected from natural riverbank, roadcut exposures or quarry (see location on Supplementary Fig. 3). They consist on fine to medium sand sampled in layers interbedded into or resting above coarser alluvial material. They were collected from cleaned faces by inserting steel tubes into the sandy layers. The tool used to insert the steel tubes is visible on the photographs. A minimum thickness of 15-20 cm of the sandy layers was generally considered.

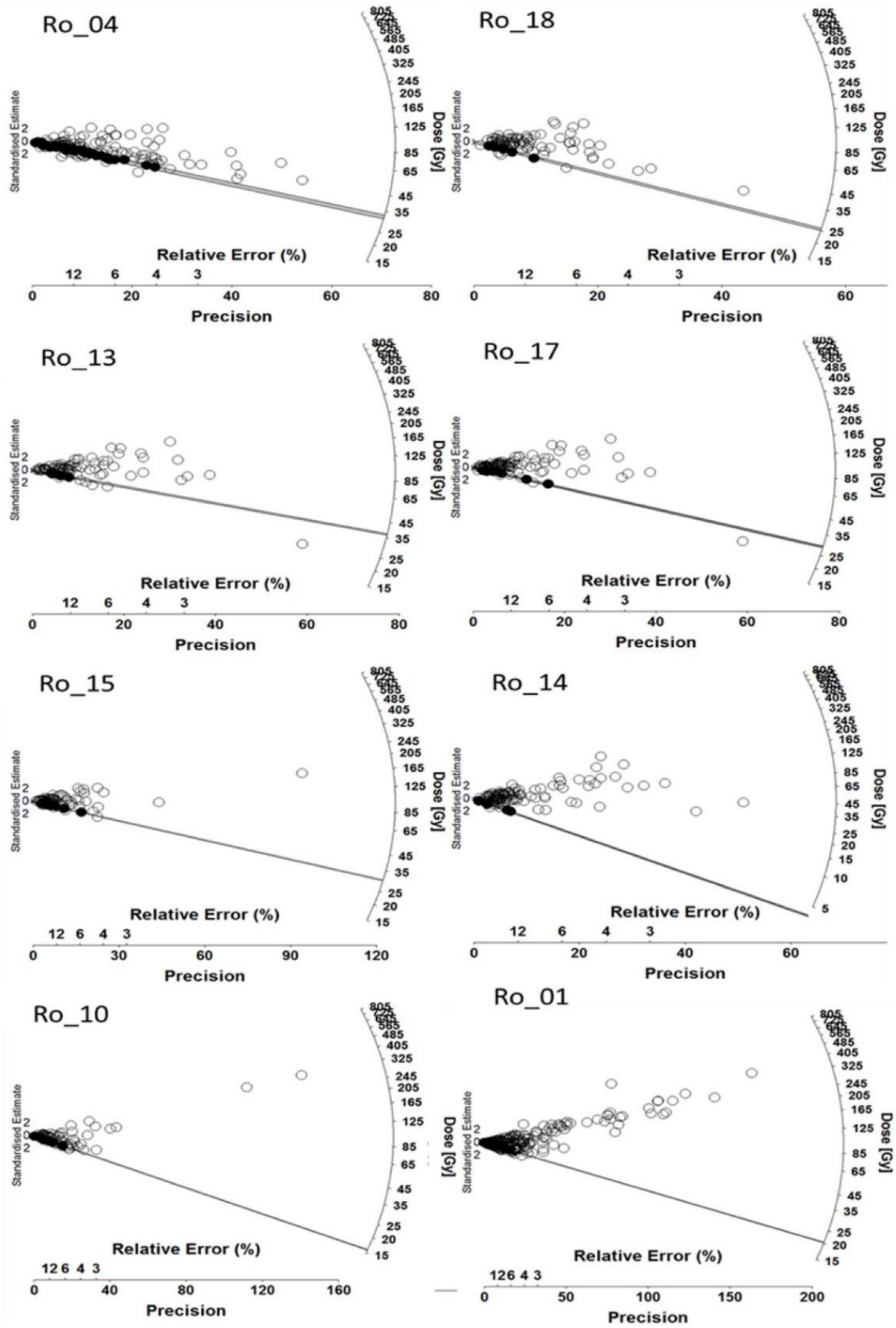

Supplementary Figure 5. Radial plots of the natural single-grain pIRIR dose distributions of the nine Rangitikei samples. The grey bar indicates the palaeodose estimate calculated with the bootstrap minimum age model (bootMAM: ref.[39]). Note: a sigma_b value of 27 ± 10 % was used as input parameter for the bootMAM.

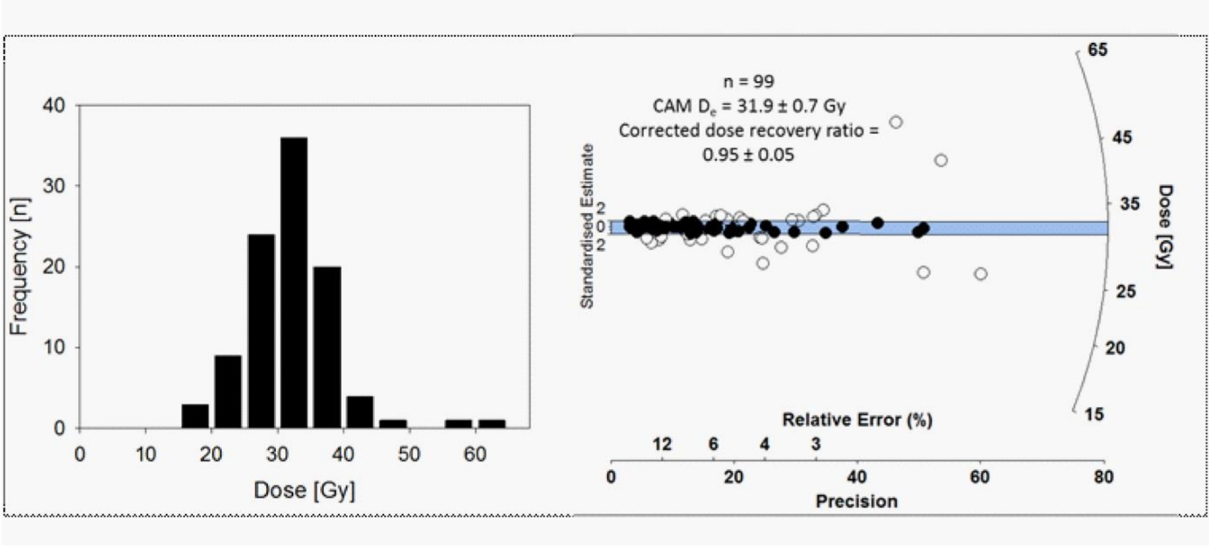

Supplementary Figure 6. Dose recovery distribution using sample RO_04. The sample RO_04 was bleached for 19 h in the SOL2 and a dose of 32.3 Gy was given. For each of the grains, the absorbed dose was then measured using the methods described above. Results are shown as histogram (left) and radial plot (right). The blue bar in the radial plot indicates the Central Age Model D_e estimate. The over-dispersion value was calculated to 18 %.

References

59. Guérin, G., Mercier, N. & Adamiec, G. Dose rate conversion factors: update. *Ancient TL* **29**, 5-8 (2011).

60. Adamiec, G. & Aitken, M.J. Dose conversion factors: Update. *Ancient TL* **16**, 37-50 (1998).
